# IS HOME HOSPICE THE “GREAT EQUALIZER?”—ADDRESSING EQUALITY AND EQUITY DURING THE COVID-19 PANDEMIC

**DOI:** 10.1093/geroni/igad104.0626

**Published:** 2023-12-21

**Authors:** Laura Moreines, Jonelle Boafo, Emily Franzosa, Patricia Kim, Dena Schulman-Green, Melissa Aldridge, Abraham Brody, Daniel David

**Affiliations:** New York University, New York City, New York, United States; Rory Meyers College of Nursing, New York City, New York, United States; Icahn School of Medicine at Mount Sinai, New York City, New York, United States; Icahn School of Medicine at Mount Sinai, New York City, New York, United States; NYU, College of Nursing, New York City, New York, United States; Icahn School of Medicine at Mount Sinai, New York City, New York, United States; NYU Rory Meyers College of Nursing, New York City, New York, United States; NYU Rory Meyers College of Nursing, New York City, New York, United States

## Abstract

COVID-19 illuminated many of the inequities that exist in the current healthcare system, especially for those who are most vulnerable and facing the end of life. Social determinants of health (SDOH) directly and indirectly influence access to the quality of care received by patients enrolled on the hospice benefit. We interviewed 20 interdisciplinary providers (nurses, managers, administrators, physicians, medical directors, social workers, and clergy) between September 2022 and January 2023 from three healthcare organizations that provided hospice care to older adults and their caregivers in NYC regarding their perceptions of the impact of SDOH on the delivery of hospice care during the pandemic. We then conducted rapid analysis of their responses. Findings emerged around three salient themes: 1. “I provide equal care to everyone” (Some providers recognize SDOH result in inequities within hospice, whereas others perceive hospice as an equalizer of care); 2. “They’re going to kill us” (Describing barriers that challenge hospice enrollment and continued access to care); and 3. “We’ve got to do better” (Addressing inequity through organizational and community programs that address barriers and improve access to hospice). Overall, we learned that SDOH must be addressed in a proactive rather than a reactive way to ensure hospice access. Systemic, rather than individual approaches were needed to overcome existing barriers exacerbated by the pandemic. To this end we will discuss lessons learned and strategies for program development and equitable delivery of home hospice care.

